# Supplementary material for: The Construction and Exploration of a Comprehensive MicroRNA Centered Regulatory Network in Foxtail Millet (Setaria italica L.)
Source: Front Plant Sci. 2022 May 6;13:848474. doi: 10.3389/fpls.2022.848474 (PMC9121102; doi:10.3389/fpls.2022.848474)
Supplement: Supplementary file 7 [file Data_Sheet_7.zip › Sit-miR399a_Seita.2G412100.1_11151_TPlot.pdf]

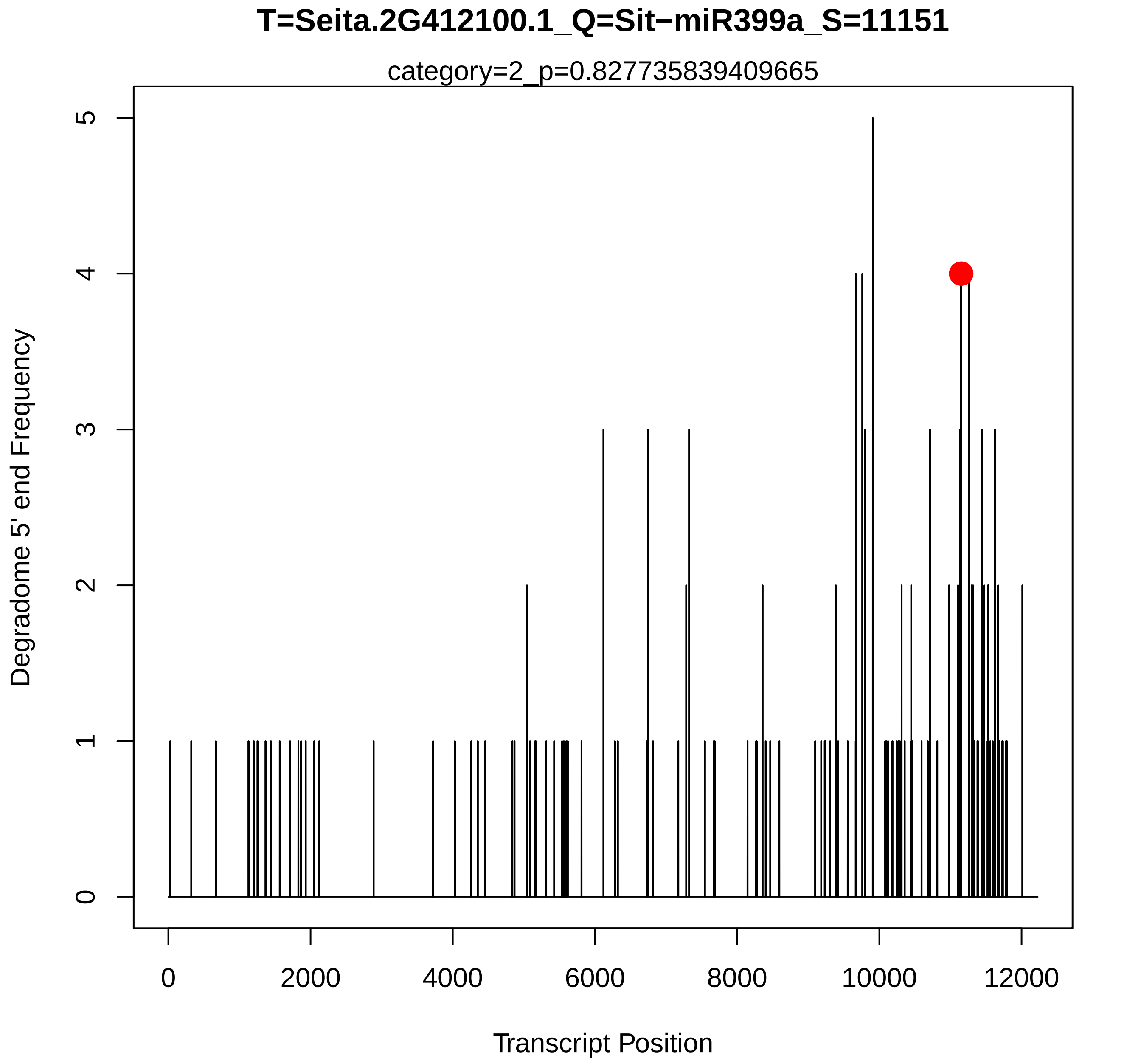
T=Seita.2G412100.1_Q=Sit−miR399a_S=11151
category=2_p=0.827735839409665
Degradome 5' end Frequency
0
1
2
3
4
5
0
2000
4000
6000
8000
10000
12000
Transcript Position
